# Stakeholders’ Initial Experience With Telemedicine Services Introduced at 13 Government Medical Colleges in Uttar Pradesh, India During the COVID-19 Lockdown: A Qualitative Study

**DOI:** 10.7759/cureus.41269

**Published:** 2023-07-02

**Authors:** Manish Singh, Abhimanyu S Chauhan, Ritika Mukherjee, Priyanka Pawar, Divita Sharma, Ahmed Shammas Yoosuf, Bharathi Vaishnav, Shikha Nargotra, Kavita Rajesh Gudibanda, Archisman Mohapatra

**Affiliations:** 1 Department of Community Medicine, Dr. Ram Manohar Lohia Institute of Medical Sciences, Lucknow, IND; 2 Department of Programs, Generating Research Insights for Development (GRID) Council, Noida, IND

**Keywords:** telehealth, remote consultation, mobile health, mhealth, ehealth

## Abstract

Background: India went into a stringent lockdown in response to the coronavirus disease 2019 (COVID-19) pandemic in March 2020, and routine outpatient and elective health services were suspended. Thus, access to healthcare services got significantly disrupted. To mitigate the impact, 21 state-owned medical colleges in Uttar Pradesh, the most populous and among the most resource-constrained states in India, had to hastily launch telemedicine (TM) services. This created an opportunity to understand how stakeholders would react to such services and what initial challenges could be faced during service delivery. Through this study, we explored the experiences of stakeholders from 13 such "new-adopter" TM centres with the main objective to identify the perceived benefits and gaps related to TM services, and what "people-centric" TM services could look like going forward.

Methods: We used an exploratory-descriptive qualitative design with a constructivist paradigm. Using interview schedules with open-ended questions and unstructured probes, we interviewed 13 nodal officers, 20 doctors, and 20 patients (i.e., one nodal officer and one to two doctors and patients from each of the 13 new-adopter centres) and stopped thereafter since we reached saturation of information. We analysed the data on NVivo (QSR International, Burlington, MA) and reported the findings using the Consolidated Criteria for Reporting Qualitative Research (COREQ) checklist.

Results: The perceived benefits that were reported included non-dependency on physical contact, economic benefit, better management of patient load, and ease of access to healthcare services. The common gaps identified in the TM services were lack of physical clinical examination, impeded communication due to lack of face-to-face interaction, technological challenges (e.g., inconsistent internet connectivity and unavailability of smartphones), lack of human resources and resources to manage the TM centres, cumbersome compliance requirements coupled with unclarity on medico-legal implications, and limited awareness of services among clients. Need for adequate promotion of TM services through information-education-communication efforts and frontline workers, strengthening of logistics for long-term sustainability, setting up a dedicated TM department at the hospitals, capacity building of the existing staff, reducing gaps in communication between doctors and patients for better consultation, and improved access to the prescribed medicines were some of the suggestions from different stakeholders.

Conclusion: The stakeholders clearly appreciated the benefits of TM services offered through the new-adopter centres amidst the pandemic disruptions. However, there were certain gaps and unmet expectations, which, if addressed, could improve the TM centres' performance with further people-centricity and enhance healthcare access and the popularity of system-based services. Avenues for sustaining the TM services and their efficient scale-up should be explored.

## Introduction

India contributes 21% of the world’s disability-adjusted life-years (DALY) while accounting for 17.7% of the population [1]. On March 25, 2020, this country of 1.37 billion people went into a nationwide "lockdown" [2]. Routine, elective, and outpatient health services were put on hold to channel the scarce health resources for surge preparedness [3]. This, however, further widened the gap between healthcare services and their access by the general public. On 24th March 2020, a day before India went under lockdown, the Board of Governors (in supersession of the Medical Council of India, India’s regulatory authority for medical colleges), in partnership with NITI Aayog (India’s highest policy advisory committee) and the Government of India (GoI), released the telehealth practice guidelines. These guidelines gave directions for the potential scale-out of telehealth services as an alternative/add-on to routine services [4]. Subsequently, the public health system quickly geared up to offer remote consultation support to the population through telemedicine (TM). Many of these health facilities had to set up these services for the first time ever. For these "new-adopter" facilities, there was minimal prior experience with the feasibility of offering such services and the potential implications thereof. However, to ensure that TM systems were "intuitive" and "effective", services must focus on the specific requirements of the users, e.g., the patients, the doctors, and the system-based providers (organizer-coordinators) [5]. Inadequate knowledge of user-centric designs has been identified as a cause of poorly performing TM systems [6].

Uttar Pradesh (UP) is the most populous state in India (approximate population of 200 million; ~14.6% of India’s population) [7]. The large population size and poor health situation portend a heavy load on UP’s health system [8]. On April 26, 2020, appreciating that the suspension of outpatient services amidst the coronavirus disease 2019 (COVID-19) pandemic had led to difficulties for the patients, the Director General of Medical Education (DGME; the highest administrative authority for medical colleges in UP), Government of UP (GoUP) directed 21 state-owned medical colleges to initiate TM services [4,9,10]. Between April and May 2020, TM services were rolled out for various speciality disciplines at these centres; these were mostly colleges that had no prior experience in providing such a service and were thus "new adopters" [11]. TM services were a potential candidate for scale-up, and the 21 "new-adopter centres" in UP provided an opportunity for rich learning. To explore stakeholders’ initial experiences with TM services offered by these facilities and their suggestions for improvement, we undertook this study. Our overall aim was to generate critical insights for programme strengthening and potential scale out of TM services from government medical colleges and beyond in resource-constrained contexts but with people-centricity.

## Materials and methods

Working definition

The World Health Organization (WHO) has defined TM as “the delivery of health care services, where distance is a critical factor, by all health care professionals using information and communication technologies for the exchange of valid information for diagnosis, treatment, and prevention of disease and injuries, research and evaluation, and for the continuing education of health care providers, all in the interests of advancing the health of individuals and their communities” [12]. The WHO identifies TM as “essential services in strengthening the Health Systems Response to COVID-19” and suggests that it should be one of the alternative models for clinical services and clinical decision support [13]. In this paper, we have used the terms "telemedicine" and "telehealth" interchangeably. We have also used the terms "human-centricity", "people-centricity", and "user-centricity" interchangeably.

Study setting and design

We conducted the study at the government medical colleges that had operationalized TM centres in response to the DGME directive dated April 26, 2020. We used an exploratory-descriptive qualitative research design with a constructivist paradigm. We collected the data between August 1 and September 30, 2020. For this, we developed interview schedules based on the review of the literature [14-16], informal expert consultations, and pretesting at a centre other than those selected for the study.

Study participants

We approached the respective nodal officers of the 13 new-adopter centres and conducted in-depth interviews (IDIs) with them, in the order in which they consented to participate. Once we completed the IDI of the nodal officer at a centre, we requested him/her to provide us with a list of two doctors and two patients (preferably a man and a woman) that had attended the centre on or around the date of request. We did semi-structured interviews with these doctors and patients using interview schedules with open-ended questions and unstructured probes.

Data collection

The health-related interviews were conducted by four investigators (ASC (man), ASY (man), MS (man), and RM (woman)). We conducted the interviews over video calls (on WhatsApp and Google Meet) and on the telephone with participants with verbal consent and prior appointment at a place of their convenience (home/office). Participants were requested to be seated at a quiet place and preferably in the absence of any other member or colleague (onlooker) for the interview. Interviews with patients were conducted by an interviewer of the same gender as that of the participant using Hindi as the language of communication. Interviews with the providers (TM nodal officer and doctors) were majorly in English. Training of the interviewers in interviewing techniques was undertaken and mock interviews were carried out prior to the actual interviews under the supervision of the senior investigator (AM). A typical IDI with the nodal officer, specialist doctor, and patient lasted between 45 minutes and one hour, 20 and 30 minutes, and 15 and 20 minutes, respectively. All interviews were audio-recorded with prior consent. In addition to the interview recordings, each interviewer maintained detailed interview notes that provided us with adequate details to discuss during the daily review carried out at the end of the day’s work and plan for subsequent data collection. The interview notes also helped in identifying early patterns as well as assessing the attainment of saturation of responses. The initial list of codes (identified deductively from the review of literature) was expanded with the addition of newer codes, and refined iteratively in consensus by the team of investigators. This iterative process ensured that the data collected were grounded and rich in details, and that saturation was obtained prior to the termination of data collection.

Data management and analysis

We undertook descriptive thematic analysis of the data using a deductive-inductive approach inspired by the model proposed by Braun and Clarke [17]. After having acquainted ourselves with the data by listening to the audio-taped recordings multiple times, we listed down the major themes and codes that were obvious from these. Subsequently, audio files were directly coded (tape analysis) using the software package NVivo (QSR International, Burlington, MA) to allow further codes and categories to emerge from within the data. The direct coding (tape analysis) helped in preventing the loss of important nuances related to the use of an English-Hindi mix along with voice modulations (to communicate meaning beyond words) by the respondent at the time of the interviews; these are potentially lost when interviews are converted into transcripts for analysis. Coding was done by two investigators (ASC and RM) and coding disagreement was sorted in consultation with a senior researcher (AM). Once all the interviews were coded, we established the relationship between the major themes (concepts) and the codes using an inductive and iterative approach through mutual discussion between the three authors. To strengthen our inductive line of argument, we presented verbatim quotable quotes from the interviews; quotes in Hindi were translated to English and back-translated to Hindi to ensure validity, and presented in English for the international audience.

The analysis was undertaken in real time, i.e., while data collection was ongoing. We stopped further data collection once the team was confident that no additional information was coming up from the interviews and that a point of saturation (code and meaning) had been attained.

Quality assurance

Interviews were conducted by qualitative researchers and monitored for completeness and correctness of responses with appropriate coding of the recordings at the time of analysis. Due to inherent limitations of the interpretation of qualitative data, we held discussions with one of the TM nodal officers (participant) and two patients (participants) for client validation of our interpretations. The study followed the Consolidated Criteria for Reporting Qualitative Research (COREQ) for reporting the findings of this qualitative research study.

Ethics approval

The study protocol was reviewed and approved by the Independent Institutional Ethics Committee of Dr. Ram Manohar Lohia Institute of Medical Sciences, Lucknow, Uttar Pradesh, India (IEC No. 79/20; Ref. 859/RMLIMS/2020; dated 12/06/2020). The study was conducted in compliance with the Indian Council of Medical Research (ICMR) ethical guidelines for biomedical research involving human participants (2017) [18]. In brief, we conducted all the interactions and data recording with prior informed verbal (recorded) consent. The interactions were done over the phone at a time and in the language (English/Hindi/both) of convenience of the participant.

## Results

We collected data from 13 TM nodal officers, 20 doctors (17 men and three women), and 20 patients (eight men and 12 women). The doctors were from various specialities, including internal medicine (n = 5), orthopaedics (n = 4), dermatology (n = 3), paediatrics (n = 2), general surgery (n = 2), dental (n = 2), pulmonology (n = 1), obstetrics and gynaecology (n = 1), and neurology (n = 1).

We organized our findings under three themes. Theme I enumerated the perceived benefits of TM services during the COVID-19 lockdown times (wave I, March-August 2020, Uttar Pradesh) as highlighted by the respondents. Theme II enlisted the gaps in TM services as highlighted by the respondents. Theme III provided the stakeholders’ suggestions for improving the services of the TM centres. We considered these potential areas for improving the people-centricity of the TM services.

Theme I: benefits of TM services valued by respondents

Ease of Access to Health Services

The COVID-19 pandemic impacted the regular functioning of the health system. Outpatient and elective services were suspended, and the staff was repurposed to COVID care. The implementation of a nationwide lockdown made it further difficult for patients and doctors to commute to the health facility. The patients were also scared of visiting health facilities during the pandemic in fear of contracting COVID-19 and often delayed treatment unless the ailment aggravated beyond bearable limits. In such a situation, TM services helped in providing access to medical consultations; this was reported as a major advantage. Referral of emergency cases through the TM prescriptions also helped patients access timely treatment.

“With teleconsultation, we get an idea of where to go, what to do and what extra tests need to be done before physically visiting the hospital. This increases the chances of getting the actual treatment faster.” (Cardiovascular and thoracic surgery patient, 32 years, woman).

The majority of the doctors and patients found TM services to be very helpful in e-consultation for patients with minor ailments and follow-ups; the patients could consult from the comforts of their homes. The stakeholders reported improved follow-up of chronic conditions and post-operative and post-partum patients. Together, it helped in reducing the inertia and delay in treatment initiation. TM was also appreciated for aiding in easier and faster access to specialist consultation in comparison to the pre-pandemic times.

“The best thing is you don’t have to go to the hospital, you save time and also it reduces risks for old age patients who are unable to travel, especially for neuro patients who have lots of other allied risks.” (Neurology patient, 73 years, woman).

Elderly patients and those with disabilities preferred TM services for their convenience. It helped them in overcoming their limited ability to commute to the health facilities. Some respondents also shared that with the help of family members and neighbours, they could successfully engage in a virtual consultation, without having to physically visit the facility. Interviews with provider stakeholders reaffirmed this.

“Although the seriously ill patient still needs to come to the hospital, patients with minor illness and follow-up can be attended via telemedicine in future.” (Nodal Officer).

Increased Participation of Patients and Caretakers During the Consultation

TM services enabled the opportunity of counselling not only the patient but also his/her family members and attendants at the same time. Joint counselling by the doctor helped in addressing varied queries, explaining the diagnosis and elucidating the line of treatment to multiple people who are/may be involved in decision-making. TM also gave the opportunity of increased participation irrespective of the place of residence, i.e., even if the family members did not reside with the patient and were located across the globe, TM allowed their participation during the medical consultation. This avoided misrepresentation or loss of information from the doctor to the patient and enabled shared decision-making.

“My mother is short of hearing and is unable to walk. She is a patient of hypertension and no matter what, she will not put on her mask. She does not listen to anyone. She cannot explain anything to the doctors, nor is she able to understand what the doctor is saying. So there needs to be a family member who can explain things to the doctor. So, for us, this has been very helpful, especially during COVID times.” (Neurology, family member of patient, 73 years, woman).

Optimization of Patient Load at the Health Facility

Provider stakeholders reported an improvement in patient load management via TM centres. They informed that patient load was equally distributed in the available time via appointment-based e-consultation. Time was segregated for registration of patients and consultation, i.e., unlike the usual practice of registering in the morning and then waiting through the day for one's turn for a consultation, care-seekers could book appointments beforehand and consult the doctor as per a scheduled date and time. There was also scope for improved scheduling of consultation appointments based on the availability of doctors of the concerned speciality and the preferred timings of patients. It also helped in reducing the workload on any particular provider by redistributing consultation appointments among available doctors. The capacity of treating those patients whose health issues could be resolved virtually through TM resulted in reduced crowding at the outpatient departments (OPDs). The majority of the doctors found this viable to sustain in future for patients with minor ailments.

“TM has resulted in better management of the patients. We schedule and distribute calls as per the availability of the doctors. This helped in addressing overcrowding of the patients at the OPD.” (Nodal Officer).

“Given the current COVID-19 situation, TM is the best. Travelling is a risk. TM saves time and also reduces the risk of getting infected.” (Orthopaedic patient, 48 years, man).

All the interviewees reported that TM services helped in a significant reduction in risk exposure for COVID-19 by decreasing the need for hospital visits and in-person consultations.

TM Services Were Cost-Saving for Care-Seekers

The majority of the respondents of the study who accessed TM services reported a significant reduction in the total cost of availing of OPD services. TM was most appreciated for being time-saving, especially by people who had to travel long distances to seek health services. Many rural respondents mentioned that the medical colleges were located far away from their place of residence (beyond 50 kilometres in certain instances). The patients were of the opinion that the TM services not only saved their travel time to the facility and its allied costs but also helped them to avoid long queues. Additionally, patients (respondents) shared that due to long waiting times, at times they had to stay overnight and accrue extra expenses of stay and food. These direct costs and other indirect expenses like wage loss and opportunity costs of both the patient and the attendant(s) could also be saved through TM services. TM was, thus, deemed convenient for the financially challenged, especially in remote locations.

“I had to travel a distance of an hour to reach the health facility at Bareilly and I landed up wasting time and money in commuting. With TM, I can just be at my home and get the required consultation done. This is extremely useful and convenient for me.” (Urology patient, 33 years, man).

Improved Patient Satisfaction

The patients perceived TM services as "prompt and responsive" and reported improved satisfaction with the public health system. While pre-pandemic services at the health facilities were deemed as time-consuming and inconvenient, TM enabled timely appointments and better patient-doctor interaction. The patients said that the TM consultations, unlike usual in-person consultations, allowed them ample time to discuss their problems with the doctor, even the senior doctors. They could not only discuss the main ailment of concern but also other allied issues, unrushed. Besides, patients (especially those with gynaecological issues or seeking contraceptive advice) reported that they were at ease to communicate from the privacy of their homes (which was at times unavailable in the crowded health facilities). They could openly discuss issues which they were otherwise hesitant to share in the presence of accompanying persons during physical visits to the facility.

“The doctors at the hospital (during in-person consultations in pre-pandemic times) also do not have so much of time to listen to the patients. There you can only share the main problem, as there are too many patients in the queue. But in TM, you can discuss in detail.” (Obstetrics and gynaecology patient, 25 years, woman).

“The doctor first made a video call, but I could not receive it. So, he then made a voice call. When they finalize a date, they call up on their own. We do not have to wait for long.” (Pain medicine patient, 53 years, woman).

Opportunity to Record and Archive the Interaction Details

The patients reported that at times, they could record (audio/video) whatever was discussed during the consultation. This was deemed quite useful since many-a-times they could revisit whatever was suggested during the consultation and follow the advice in a better way. The TM coordinators reported that TM enabled easy archiving of patient-specific electronic prescriptions and other health records, e.g., diagnostic reports, maintaining an updated database, and also for an easy cross-reference between doctors across the departments (Table 1).

**Table 1:** Theme I: perceived benefits of telemedicine (TM) services during the COVID-19 lockdown

Benefits highlighted by the respondents	
Access to health services	Access to medical consultation despite lockdown. Reduced inertia and delay in treatment initiation. Easier access to specialist consultation as compared to pre-pandemic times. Ease of getting a consultation for the elderly and people with disabilities. Improved follow-up (chronic conditions, post-operative, and post-partum).
Increased participation of patients and caretakers during the consultation	Joint counselling and decision-making.
Optimization of patient load	Reduced crowding at the health facility, Better management of patient appointments. The workload can be redistributed without overburdening any particular provider, Averting the possibility of contracting COVID-19 during hospital visits and face-to-face consultations.
TM services are resource efficient	No long queues and limited waste of time. Reduction in cost (indirect and direct) - economically beneficial.
Improved patient satisfaction	Timely appointment. Patients get more time with senior doctors during consultations. Ease communication for certain gynaecological issues.
Opportunity to maintain electronic records	Recording of the consultation can be done if needed for future reference. Electronic prescriptions and other health records can be readily archived.

Theme II: gaps in TM services highlighted by respondents

Inadequate Mobilization for Service Utilization

Many of the provider stakeholders had an opinion that the community, especially the rural community, was not sufficiently appraised about the TM and the related processes to avail the service. Only a few centres reported giving multiple advertisements at regular intervals in the local dailies; nodal officers of these centres suggested that repeated advertisements had led to an increased number of consultations. However, the majority of the TM centres reported placing maiden advertisements in the local dailies, which included contact details of the hospitals, doctors of different departments, and their respective consultation timings. This, however, was deemed to have a limited impact on the mobilization of users. Providers informed that the local dailies did not have enough penetration into the remote areas, which further compromised community mobilization. Both doctors and patients reported that the information-education-communication activities related to TM consultation were very limited and the community was not well aware of the services that were being provided.

“We advertised about the TM services once in the local dailies. I got to know that other TM centres advertised about the TM centres fortnightly or weekly. I believe that the information-education-communication activities are not sufficient at present, and the public is not aware about the TM centre and their services.” (Nodal Officer).

Inadequate Administrative Preparedness

Only a few of the TM centres in the study were found to document the whole consultation (video and/or audio), but not regularly. Interactions with the doctors showed that the majority was either not aware or not much concerned about the possible medico-legal implications of virtual consultations. Considering confidentiality as one of the mandates of the medico-legal system, not much-concerted effort was found to be in practice to safeguard the same. Interactions with the nodal officers and doctors also brought forward a scenario where there was inconsistency in documentation practices and systems to manage health records across the TM centres. Only very few doctors reported their concerns related to the unavailability of any mechanism for proper documentation of the patient records, including demographic details, medical history, consultation advice, and medications prescribed. Concerns were also raised regarding the lack of standardized operational guidelines for monitoring and evaluation of TM centres. Also, the preparedness of the TM centres varied when services were launched, which further explained the inconsistency in administrative practices across the centres.

“I am not sure about the implication of medico-legal angle in the case of teleconsultation. My only (main) concern is the possibility of misdiagnosis. We don’t have proper guidelines on record keeping like patient record, audio and/or video recording, prescription sharing etc. At the moment we are dealing with a crisis situation but for the future sustainability (of the programme), there has to be a proper system in place.” (Nodal Officer).

“We are not clear about the legal issues through TM. There are always chances of court cases.” (Specialist).

Challenges With Scheduling the Appointments

Some doctors also reported that the lack of fixed time slots for each consultation often led to spilling over beyond the allocated time. This was sometimes a concern as they felt the pressure to complete all the scheduled appointments each day, but the work hours often got extended. Patients reported that in case of emergency, TM consultations were often not helpful for acquiring same-day or emergency appointments. Patients complained that getting follow-up appointments with the same doctor was a major issue in TM. Change of doctors in consecutive consultations led to hassles of repeating medical history-taking activities and sharing results of reports with the doctor. This made follow-up consultations difficult and inconvenient for both the consulting doctor and the patient.

“Yes, there is an issue. This time I had a consultation session with Dr. Y while last time I had interacted with Dr. X. Problem is that this time I was unable to consult Dr. X who was my first doctor through TM and to whom I had explained all my ailments. He had also asked me to do some medical tests. I have some queries related to that and wanted to ask him, but I am unable to get through to him.” (Pain medicine patient, 53 years, woman).

Human Resources Issues

Nodal officers and doctors shared that during the COVID-19 lockdown, the TM centres were established hastily to cater to the need of the community while OPD services stayed suspended. However, experiences since the inception suggested a requirement for an active coordinating staff who can act as the link between the doctors and patients. In the absence of such a coordinating staff, the calls were directly received by the doctors. For availing of TM services, the patients often called on the numbers at their convenience, beyond the advertised timings. This led to back-to-back incoming calls leading to numerous phone calls from the same patient, incessant call waiting alarms during ongoing consultations, and long logs of missed calls. It also led to overflowing WhatsApp messages and text messages. Staff working at the TM centres were also reported to lack orientation to the TM processes initially, because of which they were not comfortable enough with the new technological adaptation, which in turn acted as a barrier to smooth functioning.

Issues With Phone and Internet Connectivity, and Logistics

Although a pre-requisite for the successful implementation of the TM services, a common challenge widely reported across all the stakeholders was that there was an unfavourable technical environment. All stakeholders reported that the majority of the community used regular feature cell phones and not smartphones. This significantly reduced the chances of a video consultation. Sharing pictures, prescriptions, and other multimedia messages was often not possible. For those who were able to share images or make video calls, the quality of the image shared and the lack of ease of using smartphones acted as barriers. Additionally, almost all of the respondents reported disruptions during consultations due to weak internet bandwidth, except a few stakeholders who were based at or near some major town.

“Majority of the time we face connectivity issues during video consultations. We eventually have to switch to audio calls. We cannot show the visual symptoms. I am not sure if the connectivity issue is at our side or at the hospital’s end.” (Obstetrics and gynaecology patient, 25 years, woman).

Interactions with the stakeholders also showed that some of the community members also resisted or hesitated in adapting to the "new" digital technology. This was more common amongst the elderly population. E-consultation was thus mostly limited to voice calls. However, due to poor network and connectivity issues, disruption in voice calls was also reported.

At the level of the TM centre, many nodal officers reported inadequate data packages, weak bandwidth of the internet connection, unavailability of equipment like high-definition cameras, and absence of a robust TM software or platform. Nodal officers shared that the "makeshift" arrangements could be acceptable only for the duration of the COVID-19 crisis but for the future sustainability of regular e-consultation, a dedicated TM department should be set up.

“There are many patients who don’t have a phone…. leave aside, smartphones. Most of the patients are from very poor backgrounds.” (Specialist).

“Major problem is unavailability of the internet bandwidth required for successful video consultation. At some places, there is no connectivity even for normal calls. Unavailability of the internet is not limited to the community; our department also has limited internet connectivity with issues. These issues need to be addressed first.” (Nodal Officer).

Inadequate Confidence With the Quality of Services Provided and Received

While TM was hailed as beneficial by patients suffering from minor ailments, the perception was often different for those with serious illnesses. In the latter case, people did not want to risk or delay care seeking and preferred to physically visit the OPD for consultation. The majority of the doctors too shared their concerns related to the telephonic consultations. Nearly all informed that dealing with some standard health issues, minor ailments, or follow-up consultations was found to be effective but concerns remained on the overall quality of diagnosis and safety of patients in TM. Doctors shared that during face-to-face consultations, they had the opportunity to observe a wider range of clinical signs, auscultate, measure vitals, and undertake needful physical examinations. This helped in arriving at a comprehensive and robust diagnosis, which at times went beyond what the patient had presented for. On the other hand, e-consultation being mostly audio, lacked visual cues. This became more challenging with multiple dialects in the community as it became further difficult to understand what the patient was trying to communicate. Counselling of patients was also limited. Most of the provider stakeholders were concerned about the risk of misdiagnosis and/or wrong assessment of disease severity through TM further compounded by miscommunication and in turn, leading to medico-legal issues. The doctors also expressed their concern that in TM, there was no scope for any interventional treatment unless the patient came to the facility physically.

“I don’t like to do teleconsultation. There is no physical contact between doctor and patient. We are not able to see the signs and symptoms and examine the patient. So, I don’t feel it is good way.” (Specialist).

“As I am dealing with the surgical OPD, for me it is important to do a physical examination. Without examination, it could be difficult to understand the problem. The patient tries to tell me the problem but without examination, there are doubts because according to the signs and symptoms, there are many differential diagnoses and without examination, I cannot prescribe drugs which otherwise could be harmful.” (Specialist).

A few patients complained that they did not get adequate consultation time with the doctors during the TM services and were thus unable to explain their ailments to their satisfaction. They complained that the doctors were also in a hurry as they had to consult too many patients within a limited duration. This was coupled with audibility and connection issues, which further compromised the consultation process.

“While explaining the illness often the doctor was unable to hear what I said and I too could not hear what he was saying. Thus, I had to disconnect the calls multiple times and reconnect. This took away a lot of time.” (Neurology patient, 73 years, man).

Complaints were also made about the unavailability of prescribed medicines in the local medical stores. In such cases, patients expressed that they were unsure if they should get an alternative medicine with the same combination or consult the doctor again. Even after e-consultation, the patients at times had to go to the hospital to buy the medicines. Physicians also reported that the unavailability of prescribed medicines led to issues of non-adherence to treatment and client dissatisfaction.

“I have my own medicine store. Still, I could not procure the medicines prescribed to me! How would I be able to help others who faced the same issue?” (Cardiology patient, 45 years, man).

Some of the patients perceived that some doctors assigned for TM consultations often lacked experience or were junior doctors who could not live up to their satisfaction. Patients reported that the attitude of such doctors was casual and that they lacked the capacity to provide an appropriate diagnosis and advice over TM sessions. The patients did not feel confident about the treatment protocol prescribed and felt the need to have a second opinion.

“I felt while talking that it was a junior doctor and not a senior doctor. He told me that if I have further problems, I should get a consultation done by a senior doctor in the OPD. If consultation is being provided it should have been done by a senior doctor, who is more experienced. One can understand (the seniority) when doctors interact.” (Dermatology patient, 61 years, woman) (Table 2).

**Table 2:** Theme II: perceived challenges related to telemedicine (TM) services

Challenges highlighted by the respondents	
Inadequate mobilization for service utilization	The community was inadequately aware of TM services. Passive efforts at information-education-communication for mobilization.
Inadequate administrative preparedness	Ambiguity in medico-legal implications. Lack of standardized guidelines for operations, monitoring, and evaluation of TM centres. Inconsistent documentation practices and systems to manage health records. Not all TM centres were equally prepared when services were launched.
Challenges with scheduling the appointments	Clients face difficulty in obtaining same-day or emergency appointments. Change of doctors in consecutive consultations leads to difficult follow-up. Lack of fixed time slots led to consultations beyond the allocated time (pressure on doctors).
Human resources (HR) issues	Staff inadequately oriented to TM processes. Lack of dedicated TM coordination staff. High load of incoming irrelevant messages on WhatsApp etc. Excessive load of incoming calls.
Issues with phone and internet connectivity, and logistics	Client-side issues: Poor phone and internet network connectivity. Lack of smartphones for video consultation and multimedia messaging. Inability to adapt to digital technology. Provider side issues: Inadequate data packages. Low internet bandwidth (TM centre). Unavailability of high-definition cameras. No proper TM software or platform was used.
Inadequate confidence in the quality of services provided and received	Lack of physical interaction with patients. Risk of misdiagnosis and/or wrong assessment of disease severity. Risk of missing signs and symptoms of other comorbidities. Limited scope for intervention. Inadequate client satisfaction. Difficulty in communication and counselling. Limited consultation time. Difficulty communicating with people from rural communities. Unavailability of prescribed medicines in the local pharmacy.

Theme III: respondents’ suggestions for improving TM services with people-centricity

Table 3 enlists the suggestions for improvement from the respondents (Theme III).

**Table 3:** Theme III: stakeholders’ suggestions for improving telemedicine (TM) services

Stakeholders’ suggestions to improve TM services	
Need for proper information-education-communication (IEC) through frontline workers and the promotion of TM in the community	Need for extensive IEC campaigns on TM services and its benefit through frontline workers. Integrating different mediums like local radios, skits, newspapers, and social media to disseminate information and enable penetration into the community. Repetition of advertisement at regular intervals.
Strengthening the logistics of the TM department for long-term sustainability	Adopting a dedicated, medico-legal-compliant software platform with mechanisms for detailed recordkeeping. Installing high-definition equipment and camera. Ensuring availability of an internet network with high bandwidth and uninterrupted electricity. Adapting International Classification of Diseases (ICD) for monitoring and reporting of diseases. Practising appointment-based consultation only for better management of consultation time. Involving a minimum number of intermediaries in the system to avoid data leakage. Implementing a paid service of TM to avoid prank callers.
Capacity building of the existing staff	Improving awareness and training of staff and frontline workers on TM services. Mechanisms of supportive supervision and handholding at regular intervals. Orient doctors and other staff of TM centres about the context of that community.
Setting up a dedicated TM department at the hospitals	Setting up a dedicated TM department with dedicated and qualified TM staff. Setting a system to follow up with patients regarding the availability of medicine, and completion of the treatment course. Providing an assistant to the doctors to help them in the processes and coordination.
Increase availability of TM consultation appointments	Making TM services available every day to improve its service reach. Assigning fixed time slots for each patient and informing them in advance.
Setting up a remote satellite centre at the village level	Setting up a dedicated TM department with dedicated and qualified TM staff. Setting a system to follow up with patients regarding the availability of medicine, and completion of the treatment course. Providing an assistant to the doctors to help them in the processes and coordination.
Reduce gaps in communication between the doctor and the patients for better consultation	Clear instruction and guidance to the patient who calls for the service, so that s/he does not face any difficulty in adopting the new technology. Explanations be done in simple language so that it is understandable by all alternative numbers besides video calls only through one platform should be made available.
Availability and procurement of prescribed medicines	Ensuring that prescribed medicines are available locally. Provisions for a triaging facility for patients who come for a check-up after teleconsultation.

## Discussion

We conducted this study in 13 of the 21 "new-adopter" TM centres in Uttar Pradesh, India. By capturing information across stakeholder constituencies, we identified the major benefits of and gaps in TM services during the first wave of the COVID-19 pandemic in India. Our explorations suggested that TM services had considerable potential to improve patients’ access to healthcare services in the public health system and improve the performance of healthcare services. However, there was a need to put formal techno-managerial systems (e.g., standard operating procedures, record-keeping and reporting formats, monitoring and accountability frameworks, and data privacy protocols) in place. Besides, there was also the need to strategically mobilize the community to increase utilization rates. Enabling ecological and population-level interventions was also needed. For example, initiatives for improving TM service awareness, internet coverage, mobile literacy, technological adaptation, and medico-legal processes were some of the suggestions and recommendations identified by this study.

The acceptance of TM services among patients has been reported to have increased during the COVID-19 pandemic [19]. A recent systemic review identified increased self-management, acceptance of technology because of the pandemic, improved access, and social support as the most common facilitators of the adoption of TM during the first year of COVID-19. Convenience, ease of use, low cost, availability of technology and technical literacy, better patient-provider communication, fast initiation of treatment, and perceived usefulness were other facilitators [14]. Our study also identified e-consultations facilitated better management of patient load at the medical colleges, especially for minor ailments and for follow-up patients. This held the potential prospect of reducing overcrowding at the OPDs going forward if TM services were sustained beyond the pandemic. A recent study among orthopaedic patients (surgical patients) concluded that TM can effectively reduce the need for physical visits to outpatient departments for follow-up. The response rate and overall patient satisfaction rates to TM were also high [20]. Even in more developed contexts, the administrative authorities have expressed that TM would benefit the community [21]. The utility of TM in overcoming the limited ability of the elderly and those with a disability to commute to the OPDs has also been found to be one of the enablers for TM services in previous studies [22]. To enable a shift to TM consultation, the service needed to be more user-friendly, prompt, and responsive.

TM is a hybrid system involving medical as well as information and communication technology (ICT) domains. In this study, all TM centres were "new" and managed by staff and faculty lacking relevant training for providing TM services. It has been reported that physicians using virtual media to provide healthcare services need formal training to guarantee outcomes similar to in-person consultations [23]. Previously published literature also reported the lack of trained technical people at TM centres as an important missing link and the need for trained and expert manpower to establish stable and continuous communication during teleconsultation [24]. The Centers for Disease Control and Prevention, in a report on expanding access to essential health services during COVID-19 using TM, cited poor internet connectivity, low cellular reception, technological illiteracy, and lack of access to gadgets as barriers to TM [25]. A systematic review on identifying barriers to TM, concluded technical literacy, further advancement in technology, and patient preference as major barriers on the part of patients. For TM providers, workflow issues, lack of data infrastructure, and interoperability were identified as major barriers [14]. In the current study, there were also limitations with regard to the quality of face-to-face interaction, majorly due to technical barriers. TM consultations were impeded by unfavourable technical conditions like unavailability of equipment, i.e., high-definition cameras, absence of proper TM software or platform, and weak bandwidth of the internet connection. At the community stakeholder level, poor internet connectivity, lack of smartphones, and unfamiliarity with their use proved to be major barriers to face-to-face video consultations. Most service providers also expressed the need for robust technical support and an active coordinating staff to facilitate the consultation process at the TM centres. Thus, one of the recommendations made in this regard by the majority of stakeholders in our study was to set up a dedicated TM centre with trained staff and advanced TM infrastructure and software, and satellite centres in remote areas to ensure penetration of services. Thus, there is a pressing need to have guidelines and specifications for technical equipment. As per Sabrina et al. [26], there exist TM guidelines in most South East Asian countries but the focus is mainly on ethical and clinical aspects, with not much discussion on the technology required to deliver services.

A significant reduction in the cost (travelling/boarding and lodging/loss of wages) of availing OPD services was another enabler for the utilization of TM in our study. The cost-effectiveness of TM has been reported to be related to (a) cost sharing, i.e., adequate patient volume and sharing of TM infrastructure amongst various clinical users; (b) effectiveness in terms of patient utility and successful clinical consultations; and (c) reduction of indirect costs [27]. As per a recent study at a multispecialty clinic in North India, reduced cost and reduced need for travel were found to be the extrinsic motivation for making use of telemedicine facilities [28]. As stated above, the community would increasingly appreciate these benefits of TM services if needful social communication and mobilization initiatives are strategically designed and implemented. A previous study on community awareness, experiences, and perceptions concluded that for TM initiatives to be successful, there needs to be greater public awareness and understanding of the potential benefits of TM. Empowering patients as partners in the delivery of health care may be an important factor in the growth of TM services [29].

One of the findings of our study was that there was ambiguity in the understanding of medico-legal implications around TM services. For example, patient-doctor communications often extended to interactions over social media platforms such as WhatsApp, etc., which have the risk of compromising professional privacy and confidentiality of medical records. Considering the limited exposure of physicians to TM and that these new centres were opened in view of COVID-19, there is a need for sensitization and training of physicians and support staff to maintain the continuity of services. The medical curriculum should include knowledge of the legal and clinical limitations of virtual care and competencies in virtual examination [23]. A recent study emphasized that medical students can be successfully integrated into telemedicine clinics and recommended early exposure to TM prior to residency [30]. With the release of the telehealth guidelines in 2020, NITI Aayog has also suggested that certification training would be mandated for the providers in India in due course, to be eligible to provide TM consultation services [4].

Our exploration has some limitations. Due to the pandemic situation, we had to rely entirely on phone calls and virtual meetings for data collection. We could not make physical visits to the TM centres though that was desirable and would have given us deeper insights. Our interactions were also limited to only a few doctor and patient profiles. Providers’ and care seekers’ perspectives may vary according to the speciality of the service provided/sought. We did not have an opportunity to review the prescriptions generated through the TM consultations. We anticipate that these prescriptions would have been atypical in format and comprehensiveness. During the course of our interactions with the participants, we came across a range of suggestions for further improvement. It was difficult to differentiate whether these were "needs", "wants", or "demands". It was also, at times, difficult to generalize the suggestions across all the centres. Some of these were quite innovative/disruptive and perhaps were a reflection of the "level of awareness" of the respondent. Nevertheless, these warrant further deliberations and consensus building.

## Conclusions

Technology adoption is expected to have a learning curve. This study highlights the "teething issues" that new-adopter centres in resource-constrained/inconsistent settings would expect to encounter as they initiate TM services. Nevertheless, there is ample promise to consider scaling up and sustaining TM services beyond the pandemic if designed with "people-centricity". A critical element of making services people-centric is that the efforts have to be iterative and considerate of the user’s feedback for incremental learning and consequent improvement. We hope that the findings of this study will be useful for academia, future researchers, and TM programme managers in resource-constrained settings to explore further and undertake informed decisions for making TM services further intuitive and effective, going forward.

## Appendices

**Table 4:** Profile of the patients interviewed for the study

S. No.	Department	Age (in years)	Gender
1	Urology	33	Man
2	General medicine	42	Man
3	Nephrology	54	Man
4	Dermatology	25	Man
5	Orthopaedics	63	Man
6	Pain medicine	53	Woman
7	Pulmonary medicine	59	Man
8	Cardiovascular and thoracic surgery	61	Woman
9	Cardiovascular and thoracic surgery	32	Woman
10	Neurosurgery	50	Woman
11	Orthopaedics	60	Woman
12	Cardiology	45	Man
13	Physical medicine and rehabilitation	43	Woman
14	Dermatology	61	Woman
15	Obstetrics and gynaecology	29	Woman
16	Neurology	73	Woman
17	Obstetrics and gynaecology	25	Woman
18	Obstetrics and gynaecology	22	Woman
19	Orthopaedics	48	Man
20	Ear, nose, and throat	39	Woman

**Table 5:** Perceived benefits and challenges of telemedicine (TM) services across stakeholders

	Patients	Nodal officers	Doctors
Benefits	Averting the risk of COVID-19 infection by avoiding crowded OPD/waiting rooms. Time and cost saving. Free service at home. Reduced risk and convenience for old age and differently-abled patients.	Availability of services during the nationwide lockdown. No long queues or crowded OPDs. Better management of patient load. Efficient management of minor and follow-up patients.	Effective management of minor and follow-up patients. Social distancing at the time of the COVID-19 pandemic. Better and open communication about gynaecological issues over the phone.
Challenges	Difficult to follow up with the same doctor. Difficult to get same-day appointments. Unavailability of the prescribed medicines locally. Not beneficial in acute and emergency conditions. Connectivity issues. Lower satisfaction as compared to face-to-face consultation.	Dissatisfaction both amongst doctors and patients due to no physical interaction. Limited smartphones and bandwidth at the user end. Lack of awareness about the TM services. Lack of proper and dedicated equipment for TM centres. Reluctance for new technology in staff. No proper operational guidelines for TM centres. Improper documentation due to the absence of a dedicated platform. Unclear medico-legal implications.	TM services are limited to only those who have access to mobile phones, especially smartphones. Possibility of compromised quality of diagnosis due to improper connectivity and no physical examination. Difficulty in diagnosis due to unclear pictures (e.g., X-ray photos). High load of incoming irrelevant messages on WhatsApp, etc. Excessive load of incoming calls. Difficulty in diagnosis for specialities like dermatology, orthopaedics, paediatrics, etc. Communicating about medication is challenging. Unavailability of prescribed drugs at patients’ locality. Medico-legal challenges.
